# Advances in Detection Technologies for Pesticide Residues and Heavy Metals in Rice: A Comprehensive Review of Spectroscopy, Chromatography, and Biosensors

**DOI:** 10.3390/foods14061070

**Published:** 2025-03-20

**Authors:** Yu Han, Ye Tian, Qingqing Li, Tianle Yao, Jie Yao, Zhengmao Zhang, Long Wu

**Affiliations:** 1 Hubei Key Laboratory of Resource Utilization and Quality Control of Characteristic Crops, College of Life Science and Technology, Hubei Engineering University, Xiaogan 432000, China; wyyx2016277@163.com (Y.H.); 13135631690@163.com (Q.L.); ytl20031225@163.com (T.Y.); 13477171153@163.com (J.Y.); maomaoz@126.com (Z.Z.); 2 Department of Biological Science and Technology, Wuhan Bioengineering Institute, Wuhan 430415, China; tianye85@whsw.edu.cn; 3 College of Food Science & Technology, Huazhong Agricultural University, Wuhan 430070, China; 4 Key Laboratory of Tropical Fruits and Vegetables Quality and Safety, State Administration for Market Regulation, School of Food Science and Engineering, Hainan University, Haikou 570228, China

**Keywords:** rice quality and safety, pesticide residues, heavy metal contamination, detection technology, food safety

## Abstract

Pesticide residues and heavy metals, originating from diverse sources such as agricultural practices and industrial activities, pose substantial risks to human health and the ecological environment. For instance, residues of organophosphorus pesticides may damage the human nervous system, while heavy metals such as mercury and cadmium accumulate in living organisms, potentially leading to severe organ damage. The contamination of rice with these pollutants has become a critical concern, necessitating the development of innovative detection techniques that are sensitive, accurate, rapid, portable, and intelligent. This review offers an in-depth analysis of the types, sources, health risks, and ecological impacts of pesticide residues and heavy metals in rice, providing a comprehensive understanding of the challenges and solutions associated with these contaminants. It further provides the fundamental principles, comparative advantages, and technical constraints of both conventional and emerging detection methodologies. These encompass traditional analytical techniques such as spectroscopy and chromatography, well-established immunoassay systems, as well as innovative biosensing technologies. This discussion is substantiated with representative case studies demonstrating their practical applications in rice quality assessment and safety testing. In addition, this review envisions future directions for the development of detection technologies, emphasizing the importance of miniaturization, multiplexed detection, integration with nanotechnology, and real-time monitoring systems. By providing a theoretical foundation for advancing food safety innovation, this review aims to contribute to the ongoing efforts to ensure rice quality and safety, protect public health, and preserve ecological balance.

## 1. Introduction

As a staple food for billions of people worldwide, rice plays a crucial role as a primary energy source for sustaining human life and serves as a cornerstone of global food security systems [1]. Its production and consumption have been steadily increasing, particularly in Asia and Africa, where it constitutes a significant portion of dietary intake [2,3]. However, rapid industrialization and changes in agricultural practices have exacerbated rice quality and safety issues, especially regarding pesticide residues and heavy metal contamination [4,5]. For instance, in the Gazipur region of Bangladesh, the soil surrounding four industrial areas has been severely polluted with heavy metals. In the textile and paints mix industrial area, soil concentrations of cadmium (Cd), lead (Pb), and nickel (Ni) reached 0.72 mg/kg, 104.20 mg/kg, and 5.02 mg/kg, respectively. In the chemical industry, soil concentrations of iron (Fe) and zinc (Zn) were 147.65 mg/kg and 11.27 mg/kg, respectively. The cloth dyeing industry had the highest soil concentration of copper (Cu) at 7.67 mg/kg, and the adjacent paddy field soil was found to be more polluted than the background soil [5]. These contaminants not only pose significant risks to human health but also disrupt the ecological balance of soil and water systems, thereby presenting urgent challenges that require immediate attention.

On the other hand, pesticides are an essential component of rice cultivation that are used to enhance yields and manage pests and diseases [6]. Commonly used pesticides include organophosphorus compounds, pyrethroids, and herbicides [7]. However, the improper or excessive use of these chemicals can result in elevated pesticide residue levels in rice. These residues contaminate soil, air, and water and may be absorbed by rice through its root system, leading to cumulative exposure and increased health risks over time. The maximum residue limits (MRLs) for commonly used pesticides in rice vary across countries and regions. For instance, in China, the MRL for methamidophos in rice is 0.20 mg/kg, while the previous standard for chlorpyrifos was 0.10 mg/kg. In Japan, the residue limit for carbendazim in rice is 5.0 mg/kg, whereas in the European Union (EU), it is 0.02 mg/kg. The EU also sets a residue limit of 0.01 mg/kg for chlorfenapyr and mancozeb in rice and other grain products. In China, the MRL for malathion in brown rice is 1.0 mg/kg [8,9]. In terms of exceedance rates, a study in Hunan Province in 2022 tested 400 batches of early rice samples from 20 major rice-producing counties across 13 cities. Among these, 22 types of pesticide residues were detected in brown rice, with a compliance rate of 98.0% and an exceedance rate of 2.0%. Eight sample batches exceeded the limits for methamidophos, imidacloprid, and thiabendazole [8].

**Figure 1:**
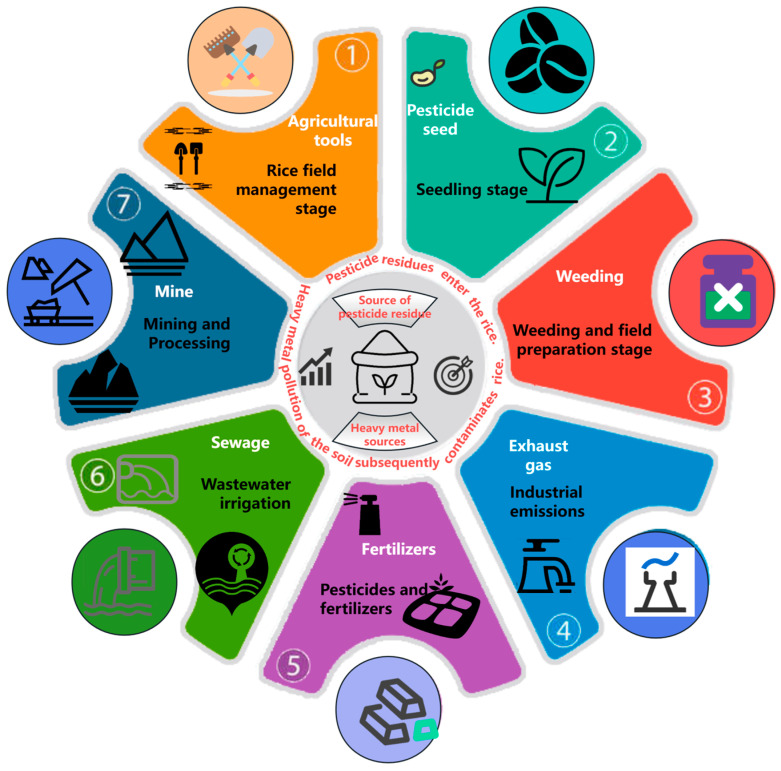
Schematic illustration of the common sources of pesticide residues and heavy metals in rice.

In addition to pesticide residues, heavy metals such as Cd, arsenic (As), Pb, chromium (Cr), and mercury (Hg) also accumulate in rice due to industrial emissions, the use of heavy-metal-containing fertilizers, and the natural composition of soil [9] (Figure 1). Regarding heavy metal limit standards for rice, China sets the maximum allowable levels for As, Cd, Pb, and Cr at 0.20 mg/kg, 0.20 mg/kg, 0.20 mg/kg, and 1.00 mg/kg, respectively, with the limit for As in paddy rice and brown rice set at 0.35 mg/kg. Internationally, the Codex Alimentarius Commission (CAC) sets the limit for As in rice at 0.20 mg/kg and for Cd at 0.40 mg/kg. The EU sets a Cd limit of 0.20 mg/kg, while Australia and New Zealand set it at 0.1 mg/kg and Japan at 0.4 mg/kg. In the Teyar River Basin of Iran, significant accumulation of heavy metals such as Cd (3.6–12.4 mg/kg) and Pb (4.9–23.6 mg/kg) has been detected in rice, posing serious health risks to local consumers [10].

Pesticide residues and heavy metal contaminants in rice not only degrade its quality and taste but also pose serious health risks [11]. Recent studies have shown that the prolonged consumption of contaminated rice significantly increases the risk of diseases such as kidney disease, neurological disorders, and cancers [12]. Heavy metals, particularly Cd and As, represent significant environmental and public health concerns owing to their potent toxicity, persistent nature, and capacity for bioaccumulation and biomagnification within ecological systems, ultimately leading to severe health risks for humans through the food chain [13,14]. The World Health Organization sets Cd exposure limits at 2.5 and 10 µg/g creatinine for environmental, occupational, and kidney damage thresholds, respectively, with β2-microglobulin below 300 µg/g creatinine. In Thailand’s Mae Sot region, over 90% of rice samples exceeded 0.4 mg/kg Cd from 2000 to 2004, leading to widespread health impacts: 50% of residents surpassed urinary Cd limits in 2009, and 60% with high Cd levels in 2004 also had elevated β2-microglobulin levels. These exposures caused 10–86% increases in kidney-related disabilities, with sex-specific risks including 12–28% higher cancer rates [15]. Emerging environmental challenges include the atmospheric transformation of pesticides into persistent toxic substances capable of long-range transport, contributing to regional ecosystem contamination. These pollutants subsequently enter terrestrial and aquatic food chains, posing significant human health risks. Additionally, the marine ecosystem faces secondary pollution threats from agricultural waste and crop residue runoff [16]. In response to these multifaceted contamination risks, the development of advanced detection methodologies for rice quality monitoring has become imperative.

Recent technological advancements have yielded significant improvements in detecting both pesticide residues and heavy metals in rice, with current analytical approaches including sophisticated spectroscopic techniques, high-resolution chromatography, sensitive immunoassays, and innovative biosensor technologies. Traditional spectroscopic techniques, such as fluorescence detection, Raman spectroscopy, and near-infrared spectroscopy, are widely used for the rapid detection of pesticide residues in rice [17]. These non-destructive, highly sensitive methods have proven effective in rice quality testing, allowing for the initial screening of pesticide residues within a short timeframe. However, these spectroscopic methods face limitations in detecting trace levels of residues and can be significantly influenced by interfering substances. Chromatographic methods such as high-performance liquid chromatography (HPLC), gas chromatography (GC), and their coupled mass spectrometry approaches (HPLC-MS and GC-MS), offer high accuracy in quantifying heavy metal and pesticide residues. They enable the precise separation and qualitative analysis of complex samples, providing reliable data for the comprehensive detection of multiple pollutants [18]. However, their high equipment costs and long analysis times restrict their practical use in field testing. The development of chromatographic separation methods depends on manual or automated chromatographic systems to conduct a series of experiments. However, due to the sequential nature of these experiments, the use of relatively large columns, and the need for significant sample quantities, screening the experimental space is costly [19].

Emerging detection methods, such as nano-immunoassay and biosensor technologies, show significant promise for on-site monitoring and rapid food safety detection. While conventional immunoassays, including enzyme immunoassays (ELISA) and fluorescence-based methods, face limitations such as lengthy processing times, high costs, technical complexity, and sensitivity constraints, their nano-modified counterparts offer enhanced sensitivity and scalability for large-scale screening applications [20,21]. Compared to traditional methods like HPLC-MS and GC-MS, which are constrained by high costs, specialized equipment, and trained personnel [22,23,24], biosensors offer more economical alternatives in food safety detection, including high sensitivity, rapid response times, and cost-effectiveness, making them particularly suitable for on-site and real-time monitoring applications [25]. Their ability to leverage specific biological interactions enables precise target recognition, while their compact design and operational simplicity facilitate field deployment and large-scale screening.

Given the critical role of rice in global food security and the escalating challenges posed by pesticide residues and heavy metal contamination, this review comprehensively examines current detection technologies and their applications in ensuring rice quality and safety. We comprehensively analyze the principles, advantages, and limitations of both traditional methods (spectroscopy and chromatography) and emerging technologies (nano-immunoassays and biosensors), with a particular emphasis on their practical implementation in field settings. By evaluating recent technological advancements and their potential to address existing detection challenges, this review aims to provide a scientific foundation for developing more efficient, accurate, and cost-effective solutions to safeguard rice quality, protect public health, and promote sustainable agricultural practices.

## 2. Types and Sources of Pesticide Residues and Heavy Metals in Rice

### 2.1. Types of Pesticide Residues

**Figure 2:**
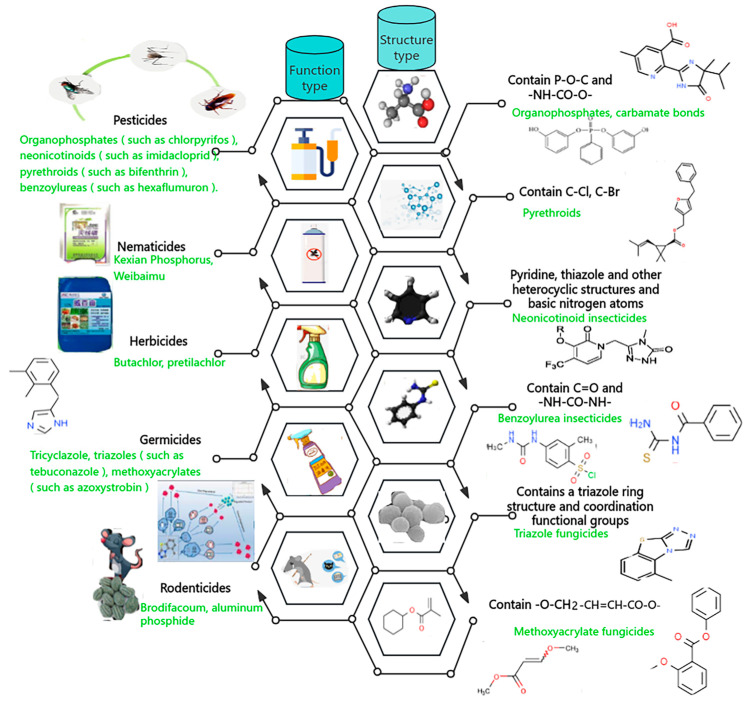
Schematic classification of pesticide residues based on their functions and chemical structures.

This section examines the health risks, ecological impacts, and scientific findings associated with various pesticide categories, focusing on their classification, mechanisms of action, and environmental consequences. Pesticides are vital for achieving optimal agricultural yields; however, their unintended adverse effects on human health and ecosystems require careful consideration. From 1996 to 2020, the global adoption of genetically modified (GM) insect-resistant and herbicide-tolerant crop technologies resulted in a 7.2% reduction (7.486 million kg less) in pesticide active ingredient usage compared to conventional farming. Based on the Environmental Impact Quotient, the environmental impact of insecticide and herbicide use declined by 17.3%. Specifically, in GM herbicide-tolerant soybeans, maize, and cotton, herbicide active ingredient usage decreased to varying extents, with the environmental impacts improving by 12.5%, 7.8%, and 6.9%, respectively. In GM insect-resistant maize, cotton, and soybeans, insecticide active ingredient usage for pest control decreased, leading to environmental impact improvements of 45%, 34%, and 17.8%, respectively [25]. Extensive research has explored the implications of pesticide use, emphasizing their potential risks in real-world agricultural applications (Figure 2).

Pesticides are classified based on their target organisms and chemical structures, with major categories including insecticides, nematicides, herbicides, fungicides, and rodenticides, each exhibiting distinct modes of action and environmental behaviors [26]. Insecticides, comprising organophosphates (e.g., chlorpyrifos), neonicotinoids (e.g., imidacloprid), pyrethroids (e.g., bifenthrin), and benzoylureas (e.g., flufenuron), primarily disrupt insect nervous systems through enzymatic inhibition or receptor interference [27]. Nematicides like clethodim and metham sodium target nematode metabolic and reproductive systems, while herbicides such as butachlor and prochloraz inhibit weed growth through specific biochemical pathways [28]. Fungicides, including tricyclazole and tebuconazole, combat fungal diseases by disrupting cell metabolism, and rodenticides like bromacil induce lethal toxicity in rodents through nervous system disruption.

From a chemical perspective, pesticides are further categorized based on their molecular structures and functional groups. Organophosphates and carbamates, characterized by phosphorus ester and carbamate bonds, respectively, inhibit acetylcholinesterase activity, disrupting neural signaling [29]. Pyrethroids, featuring halogenated hydrocarbon bonds and aromatic rings, induce toxicity by altering the ionic balance in insect nerve cell membranes [30]. Neonicotinoids, containing pyridine and thiazole heterocycles, mimic acetylcholine to impair nerve signaling, while triazole-based fungicides inhibit fungal growth through metabolic disruption.

These chemical classifications not only determine pesticide efficacy but also influence their environmental persistence and ecological impacts. For instance, pyrethroids are known for their neurotoxic effects, while organophosphates like dichlorvos demonstrate environmental persistence [31]. Herbicides have been linked to soil and water contamination, and fungicides like Paddy Mace play crucial roles in controlling rice blast disease [32]. The fumigant sulfuryl fluoride, while effective, poses significant risks to non-target organisms, highlighting the complex trade-offs in pesticide use and management.

### 2.2. Health and Ecological Risks of Pesticide Residues

**Figure 3:**
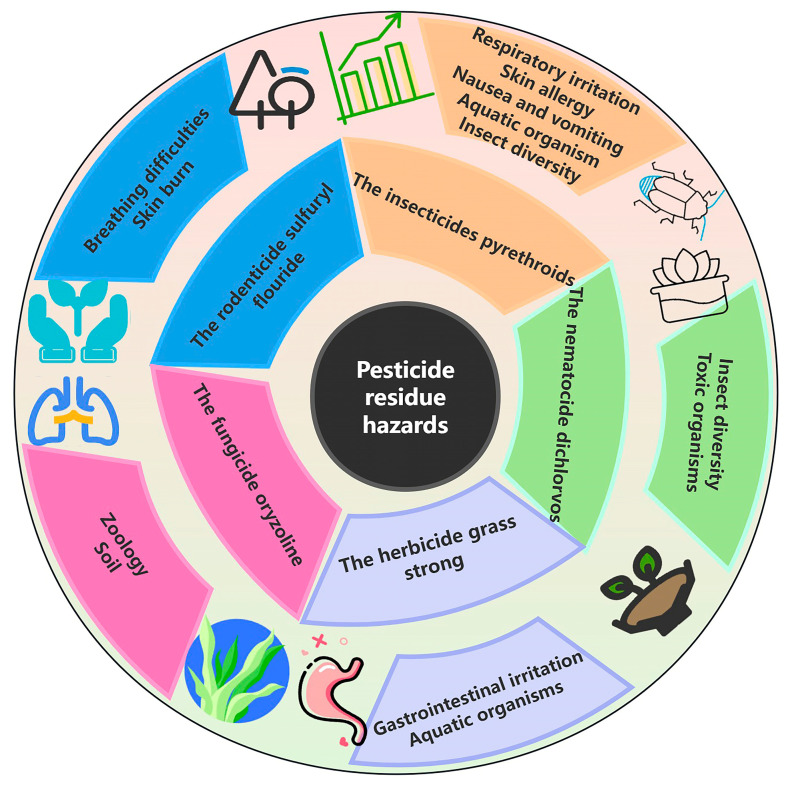
Schematic diagram illustrating the types of pesticide residues in rice and their associated risks.

Pesticide residues in rice pose significant threats to both human health and ecosystem integrity, with their impacts varying according to their chemical properties and exposure patterns. Pyrethroids, despite their widespread use, demonstrate substantial health risks through potential skin sensitization, respiratory irritation, and neurological damage upon exposure. Their ecological impacts are particularly pronounced in aquatic environments, where high hydrophobicity enables persistent sediment accumulation, leading to increased mortality among aquatic invertebrates [33]. Recent studies have revealed that stream macroinvertebrates exhibit greater sensitivity to pyrethroids than previously estimated [34], underscoring the need for revised environmental risk assessments (Figure 3).

Organophosphate compounds like dichlorvos present acute toxicity risks, manifesting as miosis, respiratory distress, and potential coma in cases of improper use. Chronic exposure scenarios reveal more insidious effects, including nervous system impairment and organ damage, compounded by the compound’s ability to bioaccumulate through the food chain, thereby increasing carcinogenic risks in mammals [35].

Herbicides, while effective in weed control, demonstrate dual health and ecological impacts. Acute exposure can induce gastrointestinal and respiratory distress, while chronic exposure may lead to nervous system damage. Ecologically, herbicide contamination disrupts aquatic ecosystems and alters soil microbial communities, potentially compromising the nutrient cycling processes essential for ecosystem health [36].

Fungicides such as Paddy Mace, despite their relatively low toxicity profile, can cause skin sensitization and gastrointestinal irritation upon prolonged exposure. Their ecological impact is particularly concerning for beneficial insects and soil microorganisms, potentially disrupting essential ecological functions and soil health maintenance. Fumigants like sulfuryl fluoride present unique challenges due to their high volatility and broad-spectrum activity [37]. Acute exposure can lead to severe respiratory complications, while chronic exposure may result in systemic damage. Research by R. Magnusson et al. [38] has demonstrated the compound’s temperature-dependent dispersion characteristics, highlighting its potential for widespread environmental contamination and non-target organism exposure.

These diverse pesticide classes collectively underscore the complex interplay between agricultural productivity and environmental health, necessitating a careful consideration of application practices and residue management strategies to mitigate their multifaceted risks.

### 2.3. Types of Heavy Metals

**Figure 4:**
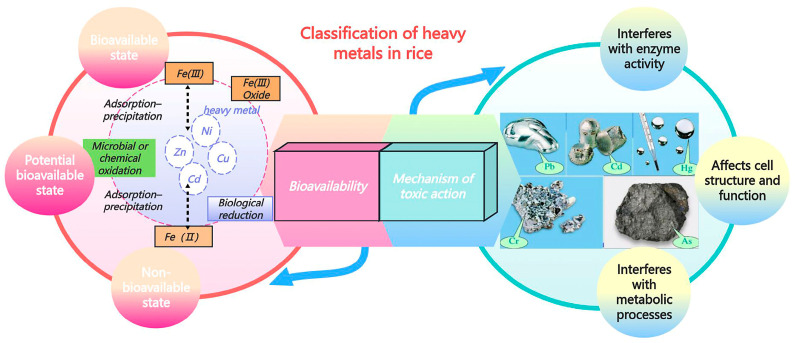
Schematic classification of heavy metals in rice based on bioavailability and mechanisms of toxic action.

This paper further explores the hazardous mechanisms of these heavy metals and their extensive ecological and health impacts. Heavy metals, originating from diverse sources, are widely distributed across various environmental media and pose severe threats to ecosystems and human health. Studies have shown that heavy metals such as Cd, As, Pb, Hg, and Cr enter the environment through multiple pathways and bioaccumulate in the food chain, posing significant risks to human health. In rice, heavy metals are categorized into three groups based on their bioavailability and toxic mechanisms of action (Figure 4).

#### 2.3.1. Classification Based on Bioavailability

Heavy metals in rice can be classified based on their bioavailability into bioavailable, potentially bioavailable, and non-bioavailable categories. Bioavailable heavy metals, such as Cd^2+^ and Pb^2+^, primarily exist in ionic forms that are readily absorbed by rice root systems, leading to their accumulation within the plant [39]. Potentially bioavailable heavy metals, including Fe and manganese, can undergo transformations into absorbable forms under specific environmental conditions [40]. Non-bioavailable heavy metals, such as Hg sulfide, generally exist as stable compounds that are not easily absorbed by rice; however, under extreme environmental conditions, these stable compounds may convert into bioavailable forms.

#### 2.3.2. Classification Based on the Toxic Mechanisms of Action

Heavy metals in rice can also be classified based on their toxic mechanisms of action. Heavy metals such as Cd and Pd inhibit enzyme activity by binding to enzyme proteins, thereby disrupting their functions. This inhibition affects processes such as photosynthesis and the antioxidant capacity, ultimately reducing the quality and yield of rice [39,41]. Hg and Cr, particularly hexavalent Cr, compromise cell structure and function by damaging cell membranes and interfering with cellular processes. For example, Cr has a negative impact on physiological metabolism [42]. This interference alters the nutritional composition of rice, posing significant health risks to humans.

### 2.4. Risks of Heavy Metals

**Figure 5:**
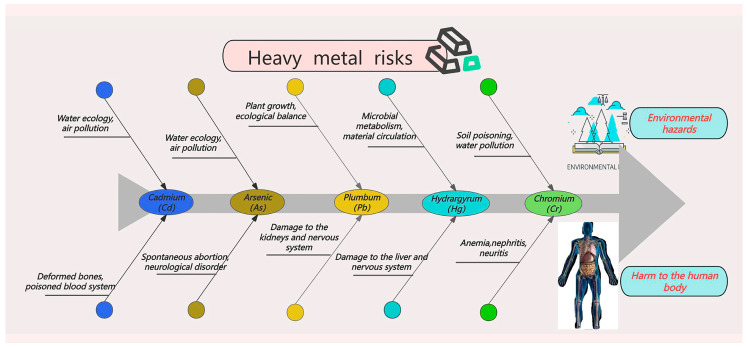
Schematic diagram illustrating the types of heavy metals in rice and their associated risks.

Heavy metals are major contributors to environmental pollution, and are ubiquitously present in water, soil, and air. These metals progressively accumulate within the food chain, posing severe and widespread threats to ecosystems and human health (Figure 5).

Cd pollution primarily originates from mining activities, electroplating processes, and the application of Cd-containing fertilizers. Its environmental impacts include reduced soil fertility, toxicity to aquatic organisms, and bioaccumulation within the food chain. Prolonged exposure to Cd is associated with renal dysfunction, bone lesions, and an elevated risk of cancer. Souza-Arroyo et al. [39] indicated that Cd, a highly toxic environmental pollutant, enters the human body through multiple pathways, accumulates, and leads to multi-organ lesions and diseases, with its hepatotoxic effects involving the disruption of metal homeostasis and the induction of oxidative stress.

As contamination arises from ore mining, the application of As-based fertilizers in agriculture, and industrial emissions. Prolonged As contamination in soil and water significantly alters their ecological functions. Acute As poisoning results in multi-organ damage, while prolonged exposure elevates the cancer risk. Studies have confirmed its harmful effects on the skin, nervous system, and liver [41].

Pb pollution primarily originates from the combustion of leaded gasoline, non-ferrous metal smelting, and various industrial activities. Pd is particularly harmful to the human nervous system, with profound implications for the brain development of children. Chronic exposure to Pd has also been associated with anemia, immune suppression, and other adverse health outcomes. Furthermore, Pd contamination in soil and water bodies disrupts the ecological balance, posing significant environmental risks [43].

Hg pollution is primarily caused by coal combustion, smelting processes, and emissions from the chemical industry. Organic Hg, which is highly toxic in nature, can severely impact aquatic organisms through biomagnification in aquatic ecosystems. Additionally, Hg can cross the blood–brain barrier, affecting the nervous system and potentially contributing to neurodegenerative diseases in severe cases [44].

Cr contamination mainly results from Cr mining and industrial emissions. Hexavalent Cr is a significant environmental pollutant, particularly in soil, groundwater, and aquatic environments, where it poses severe toxicity to aquatic organisms and soil microorganisms. Exposure to hexavalent Cr may cause skin and respiratory disorders and is classified as a carcinogen [45].

## 3. Detection Techniques for Pesticide Residues and Heavy Metals

**Figure 6:**
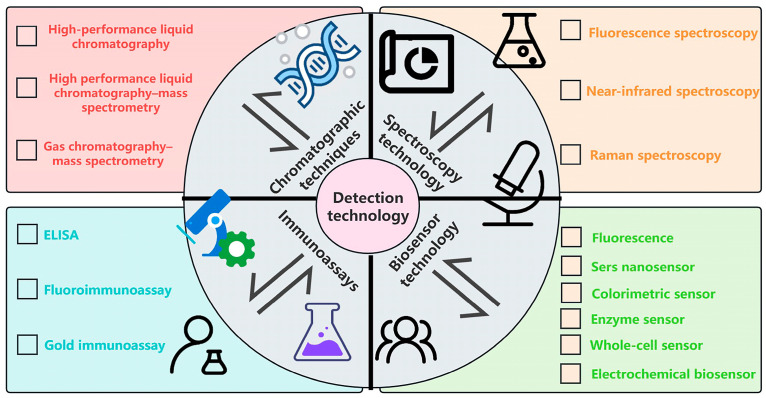
Schematic diagram of detection techniques for pesticide residues and heavy metals in rice.

Common detection methods include biosensor technology, chromatography, spectral analysis, and immunoassays. Accurate detection techniques for pesticide residues and heavy metals in rice are essential for ensuring food safety (Figure 6).

Spectral analysis techniques, including fluorescence spectrometry [46], near-infrared spectrometry [47], and Raman spectrometry [48], offer versatile detection options by analyzing the interactions of substances with light. Fluorescence spectrometry is highly sensitive but may be affected by the intrinsic fluorescence properties of the analyzed substance. Near-infrared spectrometry is a non-destructive and rapid method, although its sensitivity for detecting trace levels of pesticide residues is limited. Raman spectrometry offers detailed molecular structural insights but encounters limitations in detecting contaminants at trace levels.

Chromatographic techniques, such as HPLC [49], LC-MS [50], and GC-MS [51], separate and identify substances based on their distribution between a stationary and a mobile phase. HPLC is known for its high accuracy but requires labor-intensive sample pre-treatment. LC-MS is particularly effective for analyzing complex compounds and high-boiling-point substances, but its high cost and need for skilled operators limit its accessibility. GC-MS is user-friendly, highly sensitive, and efficient, though it is unsuitable for detecting non-volatile substances.

Immunoassay technologies, including ELISA [52], fluorescence immunoassay [53], and gold immunoassay [54], utilize the highly specific interactions between antigens and antibodies for detection. While these methods are well-suited for on-site screening, their complex preparation processes and limited resistance to interference often compromise detection accuracy.

Biosensor technologies, such as fluorescent biosensors [55], surface-enhanced Raman biosensors (SERS) [56], and enzyme biosensors [57], use biorecognition elements to convert biological signals into quantifiable data. These methods enable rapid detection and convenient applications; however, their adoption is limited by complex manufacturing processes, high costs, and insufficient resistance to interference.

### 3.1. Spectral Analysis Techniques in Pesticide Residue and Heavy Metal Detection

Spectroscopic analysis techniques, including fluorescence spectrometry, near-infrared spectrometry, and Raman spectrometry, offer distinct advantages for detecting pesticide residues and heavy metals in rice. While these techniques have been validated through practical applications, they exhibit certain limitations. Fluorescence spectrometry effectively detects substances with inherent fluorescence properties; however, its accuracy can be compromised by the variable fluorescence properties of pesticides, potentially limiting its effectiveness for certain residues. Near-infrared spectrometry enables a rapid, non-destructive analysis of contaminants; however, its lower sensitivity to trace pesticide residues renders it more suitable for detecting bulk contaminants. Raman spectrometry offers detailed molecular structural insights but encounters challenges in detecting trace contaminants due to limitations in signal intensity and sensitivity. Despite these challenges, spectroscopic techniques remain promising for food safety testing, with further advancements needed to overcome the existing limitations.

#### 3.1.1. Fluorescence Spectrometry

Fluorescence spectrometry [46] has emerged as a critical tool for detecting pesticide residues and heavy metals in rice, owing to its exceptional sensitivity and adaptability. Xia et al. [58] demonstrated its potential by synthesizing 28 chalcone-based small molecules and characterizing their physicochemical properties. Their study revealed that the nanoparticles by combining the 16-hydrogen peroxide to propylene oxide process with self-assembly characteristics of N-succinyl-chitosan (HPPO-16@NSCS) provided protective effects on rice leaves and leaf sheaths of 80.9% and 76.1%, respectively, surpassing the efficacy of the commonly used pesticide fludioxonil. Building on these findings, Yaxin Li et al. [46] developed ratio-type fluorescence biosensors based on the Ce^3+^-4-methylumbelliferyl phosphate and o-phenylenediamine (Ce^3+^-4-MUP-OPD) ternary system, achieving a detection limit as low as 0.59 ng/mL. The integration of smartphone technology further enhanced its practical application, enabling on-site visual detection with a limit of 19.6 ng/mL.

The integration of advanced computational techniques has further expanded the capabilities of fluorescence spectrometry. In a recent study, Wang et al. [59] combined three-dimensional fluorescence spectroscopy with an enhanced LeNet-5 convolutional neural network model to classify pesticides such as chlorothalonil, carbendazim, and cypermethrin. This approach achieved 100% classification accuracy, demonstrating the potential of deep learning to improve the detection precision and efficiency.

Beyond pesticides, fluorescence spectrometry [46] has proven valuable for heavy metal detection. For example, Asghar Khan et al. [60] used energy-dispersive X-ray fluorescence spectroscopy to analyze marble dust and its impact on metal accumulation in rice crops. Their findings indicated that the metal concentrations exceeded Food and Agriculture Organization of the United Nations and World Health Organization safety limits, posing health risks. Similarly, Yanrong Chen et al. [61] employed fluorescence spectrometry to monitor Cd residues in rice during a composting experiment, emphasizing the technique’s role in evaluating soil remediation effectiveness over time.

Fluorescence spectrometry [46] offers numerous advantages, including high sensitivity, rapid detection, and suitability for real-time applications. However, challenges such as weak fluorescence signals from certain substances and interference from complex matrices must be addressed. Despite these limitations, the technique remains crucial for advancing food safety and environmental monitoring.

#### 3.1.2. Near-Infrared Spectroscopy

Near-infrared spectroscopy [47] is a versatile analytical technique that detects the compositional content of substances by analyzing the transmission or reflection of near-infrared radiation. With its rapid analysis and non-destructive nature, near-infrared spectroscopy has become an essential tool in agricultural product testing, particularly for detecting pesticide residues and heavy metals, thereby ensuring food safety.

For instance, Xie et al. [47] demonstrated the potential of near-infrared spectroscopy by combining it with a back-propagation neural network model to measure the concentration of methyl isofenphos in tolfenpyrad. By calibrating and analyzing spectral signals at nine characteristic wavelengths, the study achieved correlation coefficients of 0.999 for the calibration set and 0.989 for the prediction set, with a root mean square error of 0.27%. These results underscore the precision and reliability of near-infrared spectroscopy for pesticide residue detection.

Expanding on its applications, Puttinun et al. [62] employed Fourier transform near-infrared spectroscopy coupled with partial least squares regression to develop a predictive model for monitoring rice weevil infestation in Thai Hommali rice. This approach not only quantified pest infestation but also provided insights into the relationship between insect activity and pesticide residue levels. The findings highlight near-infrared spectroscopy’s broad applicability in agricultural monitoring, offering valuable strategies for enhancing food safety control systems.

In the field of modern research, Rodrigo Cupertino Bernardes et al. [63] conducted a study using artificial intelligence (AI)-driven machine learning models. By leveraging hyperspectral reflectance data from infrared spectroscopy, they detected bee contamination with the commercial formulations of three insecticides: dimethoate (an organophosphate), fipronil (a phenylpyrazole), and imidacloprid (a neonicotinoid), as well as the commercial formulation of glyphosate, the most widely used herbicide globally. The best-performing model, linear discriminant analysis, demonstrated an impressive 98% accuracy in differentiating contamination among the bee species Apis mellifera, Melipona mondury, and Partamona helleri, with a prediction speed of 0.27 s [46,61].

Overall, near-infrared spectroscopy is notable for its speed, non-destructive nature, and versatility in diverse agricultural applications. However, challenges such as the need for advanced calibration models and potential interference from complex sample matrices warrant further investigation to fully unlock its potential for routine food safety assessments.

#### 3.1.3. Raman Spectroscopy

Raman spectroscopy is a pivotal analytical technique for detecting pesticide residues and heavy metals by analyzing the Raman scattering light generated when substances are exposed to laser irradiation. SERS [48], an advanced variant of this method, significantly enhances sensitivity by amplifying the scattering signal, establishing its critical role in food safety testing.

Notably, Xiong et al. [48] demonstrated the effectiveness of SERS by analyzing chlorpyrifos pesticide residues in rice. The study employed gold nanoparticles (AuNPs) to enhance the Raman signal and applied a successive projection algorithm (SPA) for optimal characteristic peak selection. This approach facilitated the construction of a quantitative analytical model, achieving a detection time of just 10 min with recovery rates ranging from 97.45% to 103.96%, underscoring the method’s high accuracy and efficiency. Similarly, Logan et al. [64] explored the use of a handheld SERS device to detect pesticide residues in Indian basmati rice, achieving a detection range of 5 ppb to 75 ppb. This study highlighted the potential of SERS for portable, on-site applications, offering rapid and practical solutions for food safety monitoring. Furthermore, Wang et al. [65] investigated the application of Raman spectroscopy and SERS in evaluating polylactic acid–graphene oxide composites. Their findings revealed that these materials could enable controlled pesticide release via microspheres, opening new possibilities for precision agriculture. Although Raman spectroscopy, especially SERS, has achieved remarkable results in the detection of pesticide residues and heavy metals, it still faces many challenges in practical applications. Issues such as interference from complex sample matrices and the less satisfactory sensitivity and selectivity in detecting some substances limit the efficiency and accuracy of detection. Against this backdrop, the integration of emerging technologies has become the key to improving detection performance. In the field of modern research, Diyasha Banerjee et al. [66] pointed out that AI combined with machine learning and artificial neural networks can analyze SERS data obtained from nanoparticle biosensors to achieve the accurate and rapid detection of pesticide residues in various environmental and agricultural samples. This can aid in the identification, classification, characterization, and prediction of relevant data, providing strong technical support for solving the problem of pesticide pollution. With the capabilities of AI-driven spectral analysis technology, it is expected to play a greater role in future food safety detection, contribute to solving the global problem of pesticide pollution, and push the relevant detection technologies to new heights.

The application of Raman spectroscopy, particularly SERS, in detecting pesticide residues and heavy metals offers several advantages, including exceptional sensitivity, rapid analysis, non-destructive testing, and making it highly suitable for on-site detection [48]. Despite these benefits, challenges persist. Complex sample matrices can interfere with spectral signals, while certain pesticide residues and heavy metals may exhibit insufficient sensitivity or selectivity, potentially compromising detection reliability and precision. Addressing these challenges through enhanced calibration techniques and the development of innovative materials is crucial for advancing the application of Raman spectroscopy in food safety testing.

### 3.2. Application of Chromatographic Techniques in Pesticide Residue and Heavy Metal Detection in Rice

Chromatographic techniques, such as HPLC, HPLC-MS/MS, and GC-MS, are commonly used for detecting pesticide residues and heavy metals in rice. These methods are based on distinct principles, each offering unique advantages for analytical testing, and they provide diverse options for effective and reliable detection. However, their application is not without limitations, as each technique presents specific challenges that can affect the accuracy and efficiency of detection.

#### 3.2.1. High-Performance Liquid Chromatography

HPLC [49], an advanced technique derived from classical liquid chromatography, is renowned for its excellent detection sensitivity, with low detection limits for various target substances. It also has high separation efficiency. Moreover, its excellent detection accuracy is demonstrated by strong linear relationships for various target substances, high spiked recovery rates, and low relative standard deviations (RSDs). When applied to the detection of pesticide residues and heavy metals in rice, these features make HPLC a reliable and accurate tool for analytical testing, and it has broad applicability. For instance, Raoofeh et al. [49] investigated the efficiency of diazinon (DIZ) removal from water using a carbonized product derived from rice husk mixed with polytetrafluoroethylene in a 1:2 weight ratio. The concentration of DIZ was measured using HPLC, and the study found that the optimal removal conditions occurred at pH 9, a contact time of 40 min, and an adsorbent dosage of 0.1 g/L. The results followed the Langmuir isotherm model with an R^2^ > 0.99, suggesting a strong adsorption capacity of the adsorbent material.

In another study, Vieira et al. [67] employed HPLC coupled with a diode array detector to quantify the florasulam–benzyl content in water samples. The method achieved recovery rates ranging from 95.84% to 105.4%, with RSD values under 1.5%. It demonstrated good linearity and accuracy within a concentration range of 4.00 to 150 µg/L, illustrating the robustness of HPLC in environmental monitoring.

Furthermore, Sung et al. [68] utilized HPLC combined with inductively coupled plasma mass spectrometry (ICP-MS) to measure inorganic As concentrations in rice and surrounding soil. Their findings revealed that the highest concentrations of heavy metals were found in rice samples collected near the soil, with the concentrations decreasing as the distance from the soil increased. Importantly, the inorganic As levels in all rice samples were below the safety guidelines set by South Korean regulations, ensuring that the rice remained safe for consumption.

#### 3.2.2. High-Performance Liquid Chromatography–Mass Spectrometry

HPLC-MS/MS [50] integrates the separation capabilities of HPLC with the qualitative and quantitative precision of mass spectrometry. This synergy makes it particularly well-suited for analyzing complex samples, including high-boiling-point and low-volatility substances. In the detection of pesticide residues and heavy metals, HPLC-MS/MS demonstrates exceptional sensitivity, reliability, and the ability to track transformation products and analyze trace-level contaminants.

For instance, Jia Ding et al. [50] used HPLC-MS/MS to investigate the degradation pathway of oxytetracycline (OTC) and assess its ecotoxicological impact on rice seedlings. Their findings revealed that the toxicity of OTC toward rice seedlings decreased after degradation, showing the technique’s ability to monitor chemical transformations and their ecological consequences. Elias Manca et al. [69] employed HPLC-MS/MS to study the neurotoxic effects of fipronil on the substantia nigra region of rats. By analyzing changes in VGF protein fragments, the research demonstrated a significant motor function impairment in rats exposed to fipronil, providing valuable insights into the neurotoxicity induced by pesticide exposure. In another study, Wei Li et al. [70] applied HPLC-MS/MS with an electrospray ionization source to detect tetraphenylphthalonitrile residues in various sample matrices. The method exhibited excellent linearity (R^2^ > 0.99), recovery rates ranging from 83.97% to 112.18%, and relative standard deviations between 2.58% and 15.92%. With detection limits of 1.55 to 3.09 µg/kg, this study underscored the method’s capability for trace-level analysis in food safety testing. Furthermore, Sanxiong Liu et al. [71] combined HPLC-MS/MS with Isobaric Tags for Relative and Absolute Quantitation labeling to analyze protein expression changes in rice varieties under Cd stress. They identified 61 differentially expressed proteins and mapped key pathways and proteins involved in Cd tolerance. This provided new insights into the molecular mechanisms underlying rice’s resistance to heavy metal contamination.

#### 3.2.3. Gas Chromatography–Mass Spectrometry

GC-MS [51] combines the powerful separation capabilities of gas chromatography with the precise detection and identification functions of mass spectrometry. This technique is extensively applied in food safety testing, particularly for detecting multiple pesticide residues and heavy metals. Its high sensitivity and broad detection range make it a cornerstone in analytical testing.

For instance, María Belén Medina et al. [51] utilized the “Quick, Easy, Cheap, Effective, Rugged, and Safe” extraction method in combination with GC-MS to analyze the impact of rice processing on pesticide residue levels. The study revealed that the polishing stage was the most effective at reducing pesticide residues, achieving a reduction of 66.1% to 74.7% of the initial concentrations. Additionally, dietary intake assessments indicated that pesticide residue levels in processed rice remained below the acceptable daily intake, posing minimal health risks to consumers. In another research, Mahboubeh Nozari et al. [72] applied GC-MS, ICP-MS, and a Hg analyzer to measure pesticide and heavy metal concentrations in rice samples and assess the associated health and ecological risks. Their findings revealed variations in heavy metal concentrations during different cultivation stages. While the overall health risks from consuming contaminated rice were low, the study identified nickel as a potential carcinogenic risk, underscoring the importance of targeted monitoring. Furthermore, Ihsan Ullah et al. [73] used GC-MS to investigate Cd-resistant endophytic bacteria isolated from Cd-contaminated Solanum nigrum plants. These bacteria were shown to produce indole-3-acetic acid, alleviate Cd stress in rice plants, promote plant growth, and significantly reduce Cd accumulation, highlighting their potential as a biological tool for mitigating heavy metal contamination in rice.

The practical applications of GC-MS, along with HPLC and HPLC-MS/MS techniques, underscore their crucial roles in detecting pesticide residues and heavy metals in rice [49,51]. Despite the specific limitations associated with each method, ongoing optimizations and advancements continue to enhance their efficacy. These developments are vital for ensuring rice quality and safety, supporting robust food safety frameworks, and protecting public health.

### 3.3. Immunoassays of Pesticide Residues and Heavy Metals

ELISAs, fluorescence immunoassays, and gold immunoassays play pivotal roles in detecting pesticide residues and heavy metals in food. Each method operates on distinct principles and offers specific advantages, making them valuable tools in food safety analysis.

ELISA employs solid-phase carriers combined with enzyme-labeled antigens or antibodies to achieve specific antigen–antibody reactions, producing colored products through enzyme–substrate interactions. This technique has found practical applications, such as detecting dichlorvos residues and Cd contamination in agricultural products, significantly enhancing the efficiency of routine detection processes.

Fluorescence immunoassays leverage fluorescently labeled antibodies or antigens to achieve highly accurate and stable results, particularly in spiked sample recovery tests. This method is well-suited for detecting specific pesticide residues and heavy metals, offering superior sensitivity and reliability.

Gold immunochromatographic assays [54] utilize colloidal gold as a label, providing a simple, rapid screening tool for pesticide residues and heavy metals. For instance, gold-labeled test strips have been developed for melamine detection, enabling quick and preliminary assessments of the sample status to guide further testing.

Despite these advantages, immunoassay techniques face several limitations. ELISA is often constrained by the availability and quality of high-affinity antibodies, which can introduce variability in results. Fluorescence immunoassays [53] are prone to signal interference due to environmental factors and the characteristics of fluorescent labeling. Gold immunoassays, while effective for qualitative or semi-quantitative detection, typically exhibit lower quantitative sensitivity, and their performance may decline when applied to complex matrices.

In summary, immunoassay techniques provide diverse and flexible options for food safety testing, catering to different detection needs. While their inherent limitations highlight the need for further optimization, these methods remain integral to enhancing the accuracy, efficiency, and adaptability of pesticide residue and heavy metal detection in rice.

#### 3.3.1. Enzyme Immunoassay

ELISA operates on the principle of specific antigen–antibody interactions facilitated by enzyme-labeled antigens or antibodies immobilized on a solid-phase carrier [52]. These interactions result in enzyme–substrate reactions that produce colored products, enabling a qualitative or quantitative analysis. Among the different ELISA methodologies, the double-antibody sandwich method is widely favored due to its high sensitivity and specificity. When applied to food safety testing, ELISA meets recovery rate requirements and effectively detects pesticide residues and heavy metals, contributing significantly to food safety assurance.

For instance, Jia et al. [52] addressed the challenge of detecting highly toxic organophosphate pesticides such as dichlorvos and dimethoate, which are difficult to analyze due to a lack of high-affinity antibodies. They developed a novel semi-antigen synthesis method for dichlorvos, resulting in specific monoclonal antibodies with satisfactory affinity (IC50 = 12.4 ng/mL). Using these antibodies, an indirect competitive ELISA method was developed for the simultaneous detection of dichlorvos and dimethoate. The method achieved detection limits of 12.1/14.6 µg/kg in rice, 7.3/8.8 µg/kg in cabbage, and 6.9/8.3 µg/kg in apples, demonstrating its effectiveness for a high-throughput analysis of food samples.

Similarly, Kaoru et al. [74] conducted an inter-laboratory study to assess an ELISA-based kit for detecting Cd in grains (wheat and rice) and soybeans. The study included 10 test materials and 12 blind samples, with recovery rates ranging from 84.6% to 125.1% and relative standard deviation values between 8.8% and 14.8%. These results confirmed the reproducibility of the ELISA-based kit, which performed similarly to tests for food toxins and allergens. The study highlighted the kit’s potential as a rapid, simple, and cost-effective tool for on-site Cd testing in agricultural products.

#### 3.3.2. Fluorescence Immunoassay

Fluorescence immunoassay is an advanced immunoanalytical method in which fluorescent substances label antibodies or antigens, providing a high degree of sensitivity. In spiked tests for detecting pesticides and heavy metals in food matrices, it achieves excellent recovery rates and low RSD values. Its high accuracy and stability make it a valuable tool in food safety testing. For example, Li et al. [53] developed an indirect competitive fluorescence immunoassay using quantum dots for the simultaneous detection of acetamiprid and imidacloprid in environmental and agricultural samples. The assay achieved excellent sensitivity under optimized conditions, with low half maximal inhibitory concentration (IC50) and limit of detection values, while maintaining minimal cross-reactivity. Recovery rates and RSD values across multiple sample types were highly satisfactory, and the fluorescence immunoassay results correlated well with those obtained through high-performance liquid chromatography. This highlights the potential of the fluorescence immunoassay as a rapid and sensitive method for simultaneous pesticide detection. Similarly, Sheng et al. [75] applied a fluorescence immunoassay to detect thiacloprid and etridiazole in food samples, including rice. The method demonstrated recovery rates ranging from 79.3% to 108.7%, with RSD values below 10%, showcasing its reliability and accuracy for food safety applications.

#### 3.3.3. Colloidal Gold Immunoassay

The colloidal gold immunoassay employs colloidal AuNPs as a label for antigen–antibody detection, offering both qualitative and semi-quantitative analytical capabilities. This novel immunoanalytical method is particularly advantageous for rapid screening in food safety testing, enabling the quick assessment of pesticide residues and heavy metals with simple and efficient operation [54].

As a case in point, Xie et al. [54] developed an AuNP-based immunochromatographic test strip (ICTS) for melamine detection. Under optimal conditions, the test strip achieved a visual detection limit of 40 ng/mL, with an IC50 of 4.8 ng/mL and a detection limit of 0.9 ng/mL in phosphate-buffered saline (PBS) samples when paired with a portable reader. The recovery rates for spiked fruit and vegetable samples ranged from 83.3% to 105.7%, exhibiting low variability. The results were consistent with those from indirect competitive ELISA and HPLC-MS/MS, confirming that the ICTS offers a rapid, accurate, and convenient tool for monitoring melamine levels in food. Furthermore, Jiang et al. [76] synthesized a monoclonal antibody specific to Cd^2+^ with an IC50 of 1.97 ng/mL and no cross-reactivity. This antibody was employed in a gold immunoassay test strip designed for Cd^2+^ detection in oilfield chemicals, achieving an IC50 of 1 mg/kg and a detection range of 1–20 mg/kg. The assay could be completed within 20 min, showcasing the efficiency of this method.

### 3.4. Biosensors in Pesticide Residue and Heavy Metal Detection

**Figure 7:**
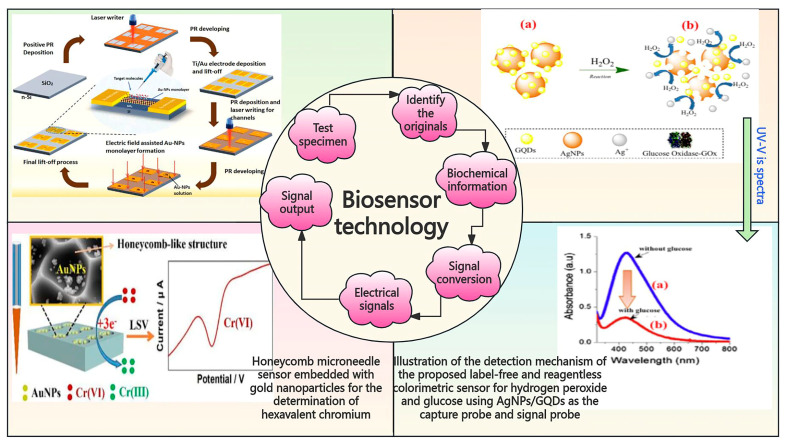
Schematic diagram of biosensors categorized according to different applications [77,78,79].

Biosensor technologies, encompassing fluorescence biosensors [55], surface-enhanced Raman biosensors [56], colorimetric biosensors, enzyme biosensors [57], whole-cell biosensors, and electrochemical biosensors, offer distinct principles and advantages for detecting pesticide residues and heavy metals in rice (Figure 7). Fluorescent biosensors function by pairing fluorophores with recognition elements and detecting changes in fluorescence characteristics (e.g., intensity, wavelength, or lifetime) caused by interactions with the target substances. These biosensors exhibit high sensitivity, a rapid response, and the ability to detect low concentrations. Surface-enhanced Raman biosensors rely on scattering effects and enhanced-signal substrates combined with recognition elements, enabling the ultra-sensitive detection and quantification of trace targets. Colorimetric biosensors detect color changes induced by chemical or biological interactions between target substances and specific reagents. They are capable of simultaneous multi-substance detection, easy to operate, and cost-effective, making them particularly suitable for field testing.

Enzyme biosensors utilize enzymes as recognition elements, detecting changes in enzyme activity triggered by interactions with target substances [57]. These biosensors offer high specificity and rapid responses, making them valuable for food safety applications. Whole-cell biosensors employ living cells to detect changes in cellular activity or gene expression caused by target substances, mimicking biological environments and providing real-time, in situ detection. Electrochemical biosensors combine biological recognition elements with electrochemical detection, leveraging changes in electrochemical signals to quantify target substances. These biosensors offer high sensitivity, fast response times, and portability, making them ideal for field and online monitoring.

Despite their advantages, biosensors face certain limitations. Fluorescent biosensors are prone to environmental interferences, while surface-enhanced Raman biosensors may experience challenges in signal reproducibility [55,56]. Colorimetric biosensors can generate false positives, enzyme biosensors often encounter issues related to stability and cost, and whole-cell biosensors require lengthy baseline establishment and costly equipment. Similarly, electrochemical biosensors may face material cost and stability constraints. Nevertheless, biosensors provide a diverse array of effective methods for detecting pesticide residues and heavy metals in rice, significantly contributing to food safety assurance.

#### 3.4.1. Fluorescent Biosensors

Fluorescent biosensors [55] operate by combining fluorophores with recognition elements, which undergo changes in fluorescence properties (e.g., intensity, wavelength, or lifetime) upon interactions with target analytes such as pesticide residues or heavy metals. These alterations are measured using specialized instruments, enabling the detection of specific substances. Fluorescent biosensors are characterized by their high sensitivity, rapid response, and ability to detect low concentrations. They can also differentiate targets through specific recognition mechanisms, making them highly effective for agricultural quality control and environmental monitoring.

Fluorescent biosensors have been extensively applied in detecting pesticide residues and heavy metals, including organophosphorus pesticides and Hg ions, where they provide accurate measurements at trace levels. For instance, Liu et al. [55] developed a porphyrin-based metal–organic framework nanoparticle biosensor for triazophos detection, achieving a detection limit of 0.6 ng/mL in rice and an 85% removal rate of triazophos. Similarly, Liu et al. [80] introduced a dual-recognition fluorescent biosensor based on triphenylamine, which effectively detected Cu^2+^ and glyphosate in environmental and biological samples. Additionally, Jin et al. [81] created a low-noise biosensor for Pb^2+^ detection, with an impressive detection limit of 77 pM.

#### 3.4.2. Surface-Enhanced Raman Scattering

SERS exploits the surface plasmon resonance effect, in which the signal is amplified through the use of substrates, such as precious metal nanoparticles [82]. When the target analyte adsorbs to the surface of these substrates, a significant signal enhancement occurs, enabling the detection and quantification of the target substance. SERS biosensors are characterized by their exceptionally high sensitivity, capability of detecting trace amounts, and ability to provide detailed structural information from the resulting spectrum. These biosensors are particularly reliable for detecting contaminants, including Cd ions and organophosphorus pesticide residues.

Several studies have explored the application of SERS for detecting harmful substances. For instance, Zhang et al. [56] developed a biosensor that integrates SERS with electrochemical methods and partial least squares (PLS) models for detecting chlorpyrifos in rice. Li et al. [83] introduced a covalent organic framework-supported catalytic amplification system for Cd ion detection, achieving a detection limit as low as 0.012 nmol/L.

Additionally, SERS has been applied in the detection of various pollutants, including organophosphorus pesticides, which are widely used in agriculture and pose significant health risks. For example, Vinod et al. [84] discussed the role of enzyme-based biosensors in detecting environmental pollutants, highlighting their reliability and specificity. This work demonstrates SERS’s ability to provide both qualitative and quantitative information on pesticide contamination.

Recent advancements in SERS technology have facilitated the development of portable, field-deployable biosensors, enabling real-time environmental monitoring. For instance, Wang et al. [85] designed a portable SERS-based biosensors for the detection of Cd ions in water, offering rapid, on-site detection with high accuracy. Such portable devices are invaluable for environmental testing, allowing for more efficient and timely pollution monitoring.

Moreover, the integration of SERS into microfluidic platforms has opened new possibilities for high-throughput screening and multiplexed detection. Chen et al. [86] demonstrated the use of a microfluidic chip integrated with SERS for the simultaneous detection of multiple contaminants, including Cd ions and organophosphorus pesticides, in water samples. This integration not only enhances sensitivity but also allows for a high-throughput analysis, making it an attractive option for environmental monitoring and food safety assessments.

#### 3.4.3. Colorimetric Biosensors

Colorimetric biosensors rely on chemical or biological interactions between the target substance and specific reagents, resulting in a color change that can either be observed visually or quantitatively measured using instruments. These biosensors are capable of simultaneously detecting multiple targets, cost-effective, and simple, making them ideal for field testing. They are widely employed for the detection of substances such as Pd ions and chlorpyrifos.

One significant area where colorimetric biosensors have been applied is in the detection of heavy metals, such as Pd ions, which are commonly found as pollutants in water and soil. For instance, Zhao et al. [87] developed a gold nanoparticle-based colorimetric biosensor for the detection of Pd ions in drinking water, achieving a detection limit as low as 30 ppb. This biosensor not only offers rapid detection but also provides a visually discernible color change, making it useful for an on-the-spot analysis without the need for sophisticated laboratory equipment.

In addition to heavy metals, colorimetric biosensors have shown promise in the detection of pesticide residues, which pose a significant threat to food safety. For example, Bordbar et al. [88] developed a paper-based colorimetric biosensors utilizing gold and silver nanoparticles for the detection of pesticide residues. Furthermore, Yang et al. [89] developed an aptamer-based colorimetric biosensor for the detection of 2,4-dichlorophenoxyacetic acid, with a detection limit of 237 nM. This biosensors demonstrated a detection limit of 237 nM, providing a simple and reliable method for pesticide detection in agricultural products.

Colorimetric biosensors have also been employed for detecting foodborne pathogens, a critical aspect of food safety. For instance, Ghazy et al. [90] developed a colorimetric biosensor using gold nanoparticles and DNA-based aptamers for the detection of Salmonella enterica, achieving a rapid and sensitive detection method suitable for the real-time monitoring of bacterial contamination in food samples.

Recent advancements in colorimetric biosensors focus on improving their sensitivity, stability, and multifunctionality. For example, Liu et al. [91] reported the development of a dual-function colorimetric biosensor that integrates both enzymatic and non-enzymatic reactions to simultaneously detect both pesticide residues and foodborne pathogens. This dual approach enhances the biosensors’ overall performance and extends its applicability to broader environmental and food safety monitoring.

#### 3.4.4. Enzyme Biosensors

Enzyme biosensors [57] utilize enzymes as recognition elements, exploiting their specific catalytic properties and the effects of target substances (such as pesticides or heavy metals) on enzyme activity. By measuring changes in signals (e.g., electrical current, potential, or light absorption) during enzyme-catalyzed reactions, the target substance can be detected. These biosensors are rapid, provide accurate results, and are highly specific, making them particularly useful for the detection of organophosphorus pesticides and certain heavy metals. For instance, Gogol et al. [92] developed an amperometric biosensor based on acetylcholinesterase, an enzyme known for its role in the breakdown of acetylcholine. This biosensor detects organophosphorus pesticide residues by monitoring changes in the electrical current resulting from enzyme inhibition upon pesticide exposure. The biosensors demonstrated high sensitivity and specificity for detecting pesticide residues in rice, with detection limits as low as 1 nM. The use of acetylcholinesterase in biosensors for pesticide detection is widely studied, as organophosphorus pesticides are known to inhibit the enzyme, leading to a measurable decrease in enzyme activity. For example, Sharma et al. [93] developed a biosensor using cellulose nanofibers derived from rice husk, which were functionalized to immobilize enzymes for the detection of organophosphorus pesticides. This biosensor integrates green chemistry principles by utilizing biodegradable materials, providing an environmentally friendly and cost-effective alternative to traditional biosensor platforms. The cellulose-based biosensor showed promising results for the detection of pesticide residues in agricultural products, offering both sensitivity and ease of fabrication.

In addition to pesticides, enzyme biosensors have shown significant potential in the detection of heavy metals, which are toxic pollutants in water and soil. For example, Elsebal et al. [94] developed an enzyme-based biosensor for the detection of Hg ions in environmental samples. By utilizing Hg-sensitive enzymes, this biosensor was able to detect Hg contamination at concentrations as low as 1.8 × 10^−11^ M, a level that is of great concern for public health. The use of enzymes in metal ion detection provides the advantages of high selectivity and rapid response, as the enzyme’s activity is directly influenced by the presence of specific metal ions. Furthermore, Wu et al. [95] reported the development of an enzyme biosensor using gold nanoparticles to immobilize glucose oxidase for the detection of glucose. The biosensors exhibited enhanced catalytic activity and stability, providing accurate glucose monitoring in medical applications. This approach could be adapted to pesticide or heavy metal detection, further enhancing the capabilities of enzyme biosensors.

#### 3.4.5. Whole-Cell Biosensors

Whole-cell biosensors utilize live cells as core sensing elements. When target substances interact with the cells or bind to their surface receptors, physiological or biochemical changes (such as gene expression or enzyme activity) occur, resulting in detectable signals, such as fluorescence or alterations in metabolic products. These biosensors mimic real biological environments, enabling in situ detection and providing valuable insights into the interactions between pollutants and biological systems. Whole-cell biosensors are particularly useful for studying the impacts of heavy metals on crops or the metabolism of pesticide residues in plants.

For example, Francisco et al. [96] evaluated the use of Ochrobactrum tritici and Nitrospirillum amazonense as whole-cell biosensors for detecting Cr(VI) in rice and maize. These bacteria were selected for their natural abilities to interact with and respond to Cr(VI), a toxic heavy metal commonly found in contaminated soil. The whole-cell biosensors demonstrated high specificity and sensitivity in detecting Cr(VI) at low concentrations, which is critical for monitoring environmental pollution and ensuring the safety of food crops. The ability to use these live cells for on-site, real-time detection makes this method an attractive option for agricultural and environmental applications.

Similarly, Lena et al. [97] assessed the interactions between As(III) and dissolved organic matter using a whole-cell biosensor. The study aimed to mitigate As absorption in plants, which is a significant concern in agricultural areas contaminated with As. The biosensor provided a useful tool for understanding how As interacts with the environment and affects plant health, particularly in regions where As contamination poses a risk to both human health and agricultural productivity. Recent advancements in whole-cell biosensors have focused on improving their sensitivity, stability, and scalability. For instance, researchers have incorporated microfluidic technology and nanomaterial-based enhancement strategies to improve the performance of whole-cell biosensors.

#### 3.4.6. Electrochemical Biosensors

Electrochemical biosensors integrate biological recognition with electrochemical techniques. These biosensors utilize a modified electrode surface to immobilize recognition elements, which interact with target substances. This interaction induces changes in electrochemical signals (such as current or potential) that are proportional to the concentration of the target substance. These biosensors are highly sensitive, rapid, portable, and ideal for field and online monitoring.

For example, Mulyasuryani et al. [98] developed an electrochemical biosensor designed for the detection of organophosphorus pesticides, which are commonly used in agriculture but pose significant health risks. The biosensor demonstrated a low detection limit of 0.18 ppm, showcasing its ability to detect trace levels of pesticides in environmental and agricultural samples. The electrochemical approach used in this biosensor allows for rapid and accurate detection, which is crucial for ensuring food safety and minimizing the risks of pesticide contamination. In a different application, Cui et al. [99] constructed a photoelectrochemical biosensor to detect the N6-methyladenosine (m^6^A) modification in RNA, a key regulatory mechanism in various biological processes. This biosensor not only demonstrated excellent selectivity and sensitivity but also provided insights into the effects of m^6^A modifications on rice, shedding light on the roles of epitranscriptomic changes in plant development and stress response. The use of electrochemical biosensors in this context is especially beneficial for plant biology research, enabling real-time tracking of molecular modifications that affect plant health and productivity. As shown in Table 1, the applications of biosensors in different fields are summarized.

Recent advancements in electrochemical biosensors have focused on improving their sensitivity, selectivity, and ease of use. One promising direction involves the integration of nanomaterials, such as graphene, gold nanoparticles, and carbon nanotubes, to enhance the performance of these biosensors. Nanomaterials can improve the surface area of the electrode, increase the immobilization efficiency of recognition elements, and facilitate faster electron transfer, leading to enhanced sensitivity and response times. For example, Sanju et al. [100] reported the development of an electrochemical biosensor based on graphene oxide for detecting pesticide residues, achieving detection limits as low as 0.01 ng/mL, a significant improvement in sensitivity.

**Table 1:** Analytical performance of biosensors for detecting different pesticide residues and heavy metals (Note: /, not reported).

Material	Linear Range	LOD	Detection time (T)/Reproducibility (RSD or CV)/Stability (S)	Real Samples	Equipment Cost	Material Cost	Reference
Fluorescence spectrometry	0.5–50 μg/L	0.05 μg/L^−1^	T = 190 s RSD = 1.9%	Rice	USD 80,000	USD 30	[101]
Near-infrared spectroscopy	1–100,000 μg/L	≤1 μg/L	/	Edible oil	USD 120,000	USD 50	[102]
Raman spectroscopy	9.99 × 10^−3^–99.9 μg/L	0.0999 μg/L	RSD ≤ 8.98%	Apple juice	USD 180,000	USD 10	[103]
HPLC	1–100 μg/L	4.5 μg/L	RDS = 3.8–13.3%	Rice, pea, wheat	USD 70,000	USD 50	[104]
HPLC-MS/MS	5–60 μg/L	0.01–0.2 μg/L	RSD ≤ 10%	Fish, tomato sauce	USD 1,000,000	USD 500	[105]
GC-MS	100–10,000 μg/L	4–780 μg/L	RSD ≤ 8%	Wood vinegar samples	USD 400,000	USD 150	[106]
ELISA	0.35–1.0 μg/L	0.03 μg/L	RSD ≤ 13%	Milk, eggs, honey	USD 10,000	USD 10	[107]
Fluoroimmunoassay	5 × 10^−5^–0.1 μg/L	1 × 10^−5^ μg/L	/	Animal-derived foods	USD 15,000	USD 90	[108]
Gold immunoassay	0.02–0.75 μg/L	0.012 μg/L	/	Shellfish samples	USD 5000	USD 5	[109]
Fluorescent biosensors	6.6 × 10^−3^–5.94 μg/L	1.06 × 10^−3^ μg/L	RSD ≤ 5.23%	Urine samples	USD 6000	USD 120	[110]
Surface-enhanced Raman biosensors	0–8.49 × 10^7^ μg/L	4.25 × 10^6^ μg/L	/	Xylene	USD 180,000	USD 10	[111]
Colorimetric biosensors	10.0–110.0 μg/L	4.7 μg/L	RDS = 1.90–5.44%	Spiked human serum samples	USD 5000	USD 20	[112]
Enzyme biosensors	1.80–1.80 × 10^5^ μg/L	0.27 μg/L	/	Human serum	USD 10,000	USD 80	[113]
Whole-cell biosensors	0.01–1 μg/mL	0.037 μg/mL	/	Human serum	USD 30,000	USD 120	[114]
Electrochemical biosensors	1 × 10^−5^–8.2 × 10^−4^ μg/L	7 × 10^−6^ μg/L	T = 45 min RSD = 3.6–9.8%	Cereal and feed samples	USD 6000	USD 30	[115]

## 4. Conclusions

This comprehensive review addresses the critical issue of pesticide residues and heavy metal contamination in rice, which pose significant threats to human health and the environment. The review provides an in-depth analysis of the sources, types, and health risks associated with these contaminants, highlighting their detrimental effects on rice quality and safety. It further explores various detection technologies, including spectroscopy, chromatography, immunoassays, and biosensors, discussing their principles, strengths, limitations, and applications in rice testing. This review emphasizes the importance of integrating these technologies to enhance detection accuracy and efficiency, thereby ensuring rice quality and safeguarding public health.

Recent advancements in detection technologies have shown promising potential, particularly in the development of biosensors that offer rapid, sensitive, and on-site detection capabilities. However, challenges remain in addressing issues such as cross-reactivity, matrix interference, and the need for standardized protocols. Future research should focus on miniaturization, multiplexed detection, and the integration of nanotechnology and data analytics to further improve the detection capabilities. Additionally, the development of portable and cost-effective devices suitable for field applications is crucial for real-time monitoring and early contamination detection.

In conclusion, the continuous innovation and optimization of detection technologies are essential for addressing the growing concerns related to pesticide residue and heavy metal contamination in rice. By bridging the gap between the current technological capabilities and future needs, researchers can contribute to the advancement of food safety standards and the protection of ecological balance. This review serves as a valuable reference for researchers, policymakers, and practitioners working towards enhancing rice quality and safety and promoting sustainable agricultural practices.

## Data Availability

No new data were created or analyzed in this study. Data sharing is not applicable to this article.
